# The impact of thermodynamics when using a catalyst for conventional carbon capture solvent regeneration

**DOI:** 10.1038/s41467-023-39694-w

**Published:** 2023-07-13

**Authors:** Frédérick de Meyer

**Affiliations:** 1 TotalEnergies S.E., OneTech, Gas and Low Carbon, CO2 and Sustainability R&D, 2 Place Jean Millier, 92078 Paris, France; 2 Mines Paris PSL University, CTP-Centre of Thermodynamics of Processes, 35 rue Saint Honoré, 77305 Fontainebleau, France

**Keywords:** Carbon capture and storage, Chemical engineering, Heterogeneous catalysis

**arising from** M. Alivand et al. *Nature Communications* 10.1038/s41467-022-28869-6 (2022)

To reduce the overall energy consumption of the solvent-based CO_2_ capture process, catalysis of solvent regeneration has been proposed by several research groups. Alivand et al.^1^ present an efficient catalyst to speed-up the desorption of CO_2_ from aqueous MonoEthanolAmine (MEA) at a lower temperature. The desorption process is, however, fundamentally controlled by thermodynamics which will limit the quantity of CO_2_ desorbed at a lower temperature. Consequently, this approach might not be beneficial if a conventional CO_2_ capture solvent like aqueous MEA is used.

Alivand et al. present a nanocatalyst to accelerate the desorption of CO_2_ from aqueous MonoEthanolAmine (MEA) at temperatures at 88 °C, much lower than commonly used in the industry (~120 °C), thus reducing the energy consumption related to solvent regeneration significantly (by 44%). However, the authors don’t explain that conventional industrial desorption of CO_2_ from aqueous amines is fundamentally controlled by thermodynamics, rather than kinetics. The high regeneration temperature in the industry is mainly required to obtain a lean solvent with a low CO_2_ loading, rather than to accelerate the CO_2_ desorption kinetics. Lowering the regeneration temperature significantly from ~120 °C to 88 °C, with or without a catalyst, should thus result in a solvent that is less well regenerated (a lean solvent with a higher CO_2_ loading) and this will negatively impact the cyclic CO_2_ absorption capacity. This will lead to higher solvent flow rates (increasing regeneration energy) and larger equipment (the opposite of a low-cost and green CO_2_ capture).

The authors justify their work in the introduction by claiming: “The remarkable energy consumption of CO_2_ separation is mainly attributed to the high solvent regeneration temperature (above 100 °C) required to accelerate CO_2_ desorption kinetics.” This statement is incorrect and therefore undermines the justification and conclusions of the work.

The aim of the industrial solvent regeneration is twofold:

1. to regenerate the loaded solvent: to obtain a lean solvent with a much lower CO_2_ loading than in the loaded solvent so that this lean solvent can be recycled back to the absorption column. The difference in acid gas loading between the lean and the loaded solvent needs to be sufficient to have an acceptable cyclic CO_2_ absorption capacity.
2. to separate the CO_2_ from the solvent so that it can be conditioned and transported for storage, enhanced oil recovery, conversion, and so on.

Therefore, in the industrial process the aqueous amine solvent is heated up to ~120 °C in the solvent regeneration column to^2^:

1. Thermodynamically favor the desorption of CO_2_. CO_2_ desorption is an endothermic process, fundamentally controlled by thermodynamics (liquid-vapor equilibrium^3^). In other words, the higher the desorption temperature, the lower the acid gas loading in the lean solvent. The impact of the temperature is significant^3^. Ideally one would like to operate the column at higher temperatures to thermodynamically favor a leaner solvent (not to increase desorption rates). However, to avoid the degradation of the amine, this is not possible. Lowering the desorption temperature, with or without a catalyst, will always result in a higher acid gas loading in the lean solvent (and thus in a lower cyclic CO_2_ absorption capacity and thus a higher solvent flow rate). Note that the maximum loading is determined by the stoichiometry of the chemical absorption reactions: 0.5 mol of CO_2_ per mol of MEA. The typical lean loading is around 0.2 mol of CO_2_ per mol of MEA. There is thus not much margin. Potential measures to improve the cyclic capacity (e.g., lowering the absorption temperature) are not linked to the use of a catalyst.
2. Generate some steam to strip the CO_2_. In the condenser the water is subsequently separated from the CO_2_ and sent back as reflux. The pure CO_2_ is subsequently conditioned.

The addition of a catalyst to speed-up the desorption and to regenerate at lower temperatures (88 °C) should thus result in much lower CO_2_ loadings in the lean solvent (see liquid-vapor equilibrium curves of CO_2_ in aqueous MEA at different temperatures^3^). In theory one could accept much lower cyclic loadings. However, applying this solution to a conventional CO_2_ capture unit with an aqueous amine solvent will result in much higher solvent flow rates (and thus an increase in the regeneration energy) and larger equipment. Not necessarily in a low-cost and green CO_2_ capture as claimed by the authors. The 44% reduction in energy consumption should thus be recalculated to compare a base case real industrial system (not aqueous MEA at 88 °C) with the catalytic system. Given the discussion above, the energy reduction will almost certainly be much lower.

All those points have been addressed in detail in a recent review in *Chemical Engineering Journal*^2^.

## References

[1] Alivand MS (2022). Engineered assembly of water-dispersible nanocatalysts enables low-cost and green CO_2_ capture. Nat. Commun..

[2] de Meyer F, Bignaud C (2022). The use of catalysis for faster CO_2_ absorption and energy-efficient solvent regeneration: An industry-focused critical review. Chem. Eng. J..

[3] Wagner M (2013). Solubility of Carbon Dioxide in Aqueous Solutions of Monoethanolamine in the Low and High Gas Loading Regions. J. Chem. Eng. Data..

